# A novel technique to integrate intraoral scans and polyvinyl siloxane impressions in situ for the completely edentulous maxilla

**DOI:** 10.1111/jopr.14055

**Published:** 2025-04-08

**Authors:** Andrew B. Cameron, Jane L. Evans, Santosh Kumar Tadakamadla, Menaka A. Abuzar

**Affiliations:** ^1^ School of Medicine and Dentistry Griffith University Gold Coast Australia; ^2^ Dentistry and Oral Health, Department of Rural Clinical Sciences La Trobe Rural Health School, La Trobe University, Violet Vines Marshman Centre for Rural Health Research, La Trobe Rural Health School, La Trobe University Bendigo Australia

**Keywords:** conventional impression, edentulous maxilla, intra‐oral scanning, novel technique

## Abstract

This dental technique demonstrates an approach to integrating an intra‐oral scan and a poly vinyl siloxane impression of the functional sulcus for a completely edentulous maxilla. The intra‐oral scan and polyvinyl siloxane impression are aligned in the oral cavity via the design and subsequent manufacture of a custom impression tray with a large window. This technique allows precise alignment of the two scans, whereas other proposed methods rely on alignment ex vivo using third‐party software which may introduce errors. The technique explained here can be applied in a variety of clinical scenarios including hyperplastic tissue, flabby or mobile ridges, or patients with sensitive gag reflexes. Moreover, it offers an alternative technique to clinicians for challenging clinical situations without the need for additional clinical appointments.

With the prevalence of edentulism predicted to continue to increase, the need for efficient/accurate prosthetic services is as important as ever.^1^ The combination of edentulism and increased life expectancy will inevitably lead to a larger population of edentulous patients and more challenging clinical situations.^1^ There has been a significant shift in the provision of removable prosthodontics with the introduction of digital techniques.^2^, ^3^ This has been driven by changes in data acquisition and manufacturing methods for complete dentures.^4^, ^5^ The use of intraoral scanners (IOS) to capture the edentulous ridge for the complete maxillary arch has been shown to be a clinically suitable method from the perspective of accuracy and patient‐centred outcomes.^2^, ^6^ The benefits of utilizing an IOS to record the denture bearing areas include improved patient experiences and increased accuracy through the mucostatic nature of the impression taking technique.^7^, ^8^ However, there are disadvantages when using an IOS for capturing the edentulous maxilla or mandible, such as the inability to use selective pressure techniques and the difficulty of capturing the functional sulcus while scanning.^6^, ^9^

The functional borders of a definitive impression for a complete denture are critical to its success.^10^ Border molding is not possible with IOS due to the nature of the impression technique which does not capture movement of the facial muscles during function.^11^ This results in clinically unacceptable deviations in the vestibules in some situations which has been shown in studies comparing IOS and PVS impressions.^8^, ^11^, ^12^ Several techniques have been suggested to improve the impression of the functional sulcus for edentulous patients when making the definitive impression using IOS.^4^, ^13^, ^14^ The implementation of scanning techniques, referred to as scan strategy, has been shown to improve the accuracy of intraoral scans but specific analysis of their effectiveness in improving the functional sulcus has not been investigated for the edentulous maxilla.^8^, ^9^, ^15^ The use of retractors to stabilize the labial and buccal vestibules has also been proposed.^16^, ^17^ The results of retraction have indicated that there is an improvement to the scans of the functional sulcus; however, there are no definitive in vivo studies that investigate the effectiveness of retraction techniques for the edentulous maxilla. The only anecdotal evidence that intraoral scans are clinically viable for the construction of a complete maxillary denture is in the form of case reports or series.^4^, ^17^

Until more evidence becomes available for the use of IOS for definitive impressions of edentulous regions, the use of silicone border molding techniques continues to be warranted. This does not, however, indicate that they should not be used in combination with IOS where the mucostatic properties of the IOS are desirable. Combining IOS and silicone impressions has been explored in several case reports.^4^, ^13^, ^14^, ^18^ The use of an open tray for the treatment of a patient with a mobile edentulous ridge or rugae in the anterior maxillary ridge is an accepted clinical technique.^19^, ^20^ Hong et al. described a technique that utilized an open tray technique to make an impression of the maxillary palate and vestibules, at the same appointment when an intraoral scan is made.^14^ The impression was scanned and then aligned to the IOS using metrology software (Geomagic Control, 3D Systems). Then, the scan of the impression and the IOS were combined. Similar techniques have also been described by Song et al. and Lo Russo et al., which all rely on software for the alignment of the impression and IOS external to the patient.

A reliance remains on direct impressions using border molding to capture the functional sulcus of the edentulous maxilla. Implementing digital workflows that are adjunct and support the outcomes of conventional techniques utilized in treating patients with difficult anatomical or clinical scenarios is imperative to patient outcomes. Although in many cases complete dentures made using IOS may be successful, capturing the anatomy and functional sulcus of a highly resorbed ridge that might have mobile tissues, will pose difficulties for the clinician. In this case report and technical note, an improved method is demonstrated that aligns and integrates different anatomical regions of an intraoral scan and a polyvinyl siloxane impression in situ. Informed written consent was obtained from the patient to publish these images for this technical report.

## TECHNIQUE

**FIGURE 1:**
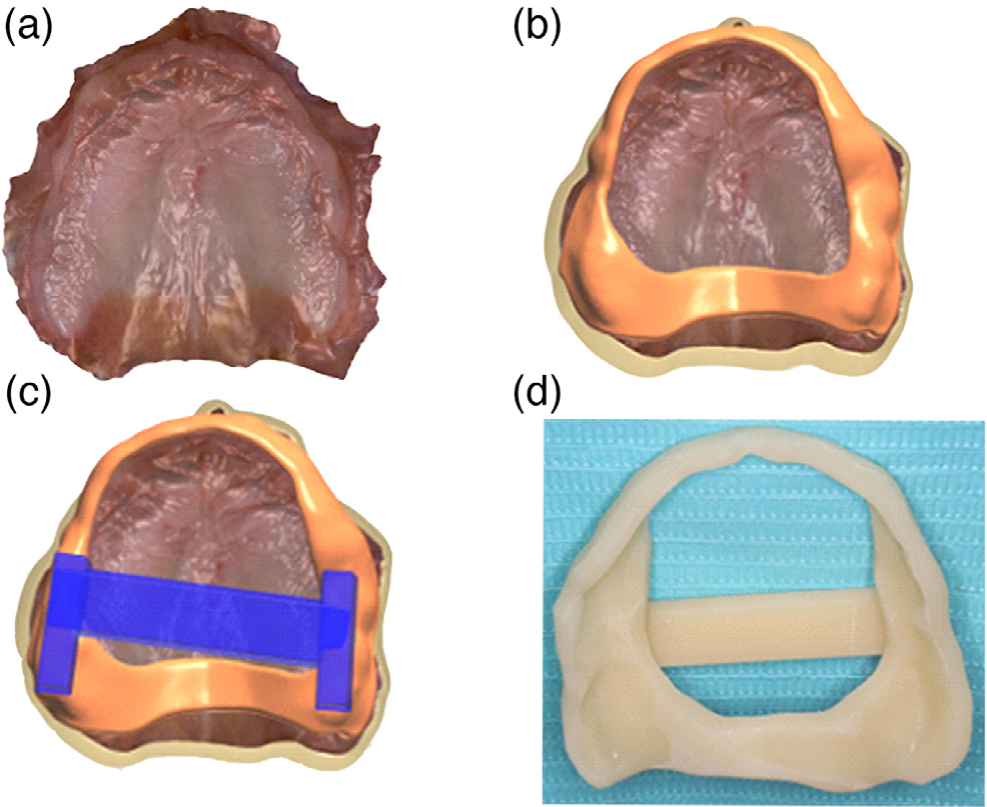
Initial intraoral scan (a). Outline of custom impression tray with open window (b). Finger stops and support bar added to custom impression tray seen in blue (c). Custom impression tray manufactured and ready for impression prior to application of adhesive (d).

**FIGURE 2:**
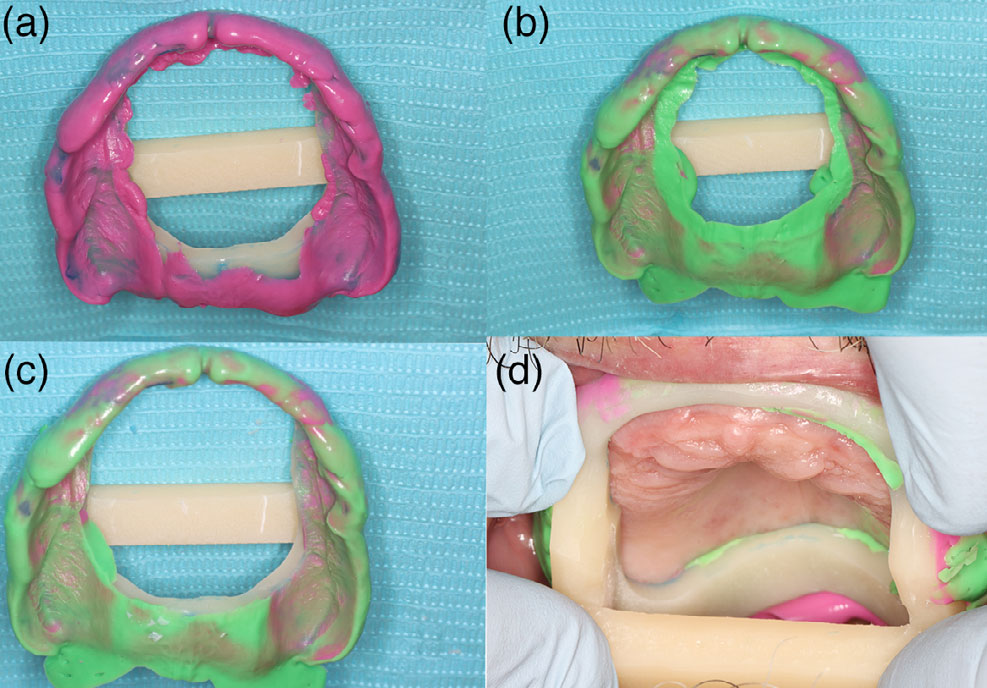
Monophase impression with border molding (a). Light body wash over border molding impression (b). Excess silicon removed with scalpel from window of impression tray (c). Window impression tray in situ (d).

**FIGURE 3:**
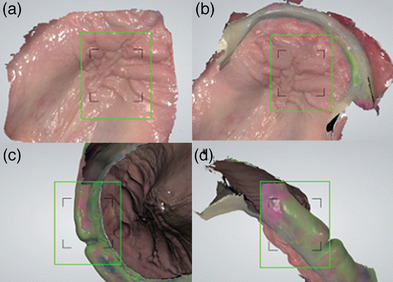
Initial intraoral scan of palatal region and rugae (a). Aligning the IOS and custom impression tray in situ (b). Scanning the intaglio peripheral region of the custom tray extra orally (c). Scanning the peripheral borders of the custom tray extra orally (d).

1. First clinical appointment: Capture the maxillary arch, with an IOS (Trios 5; 3Shape A/S) (Figure 1a).
2. Design a custom impression tray in a dental computer‐aided design program (Dental System, 3Shape A/S) that incorporates an open section on the anterior residual ridge which is bordered 10 mm anteriorly of the Fovea Palatini (Figure 1b). Include a supporting bar crossing the palate, place finger stops bilaterally in the posterior region to act as a handle (Figure 1c). The areas of the custom tray covering the post dam and hamular notch act to support the tray.
3. Manufacture the custom impression tray using additive manufacturing (Asiga UV Max, Asiga) in a photopolymer (Denta Try, Asiga) and post‐process as per the manufacturer's instructions.
4. Second clinical appointment: Assess the fit of the custom impression tray. The open section of the custom tray can be enlarged as required (Figure 1d). Make a secondary impression using a monophase impression material for border molding (Figure 2a). Make a wash impression over the border molding impression (Figure 2b), trim the excess impression material from the custom impression trays window (Figure 2c), and place the impression back in the mouth to ascertain fit and stability (Figure 2d).
5. Scan the palatal section of the edentulous maxilla (Figure 3a). Place the custom impression tray in situ and use the IOS to scan and align the intraoral scan of the mucosa to the cameo surface of the impression tray across the anterior region (Figure 3b). The peripheral region and intaglio surface of the impression can then be scanned extraorally to complete the hybrid intraoral scan and polyvinyl siloxane impression (Figure 3c,d).[Fig jopr14055-fig-0004]
6. The scanned file is then exported as a standard tessellation language (STL) and imported into an open source 3D modelling software (Meshmixer V 3.5, Autodesk, San Francisco). Sections on the scan that are not clinically relevant are highlighted and removed. The intraoral scan section of the STL is selected and “normal flipped” so that the two active surfaces of the IOS and PVS align. The edges of the two scans are then zipped together and then bridged to make a final STL that can be used as the definitive model.

**FIGURE 4 jopr14055-fig-0004:**
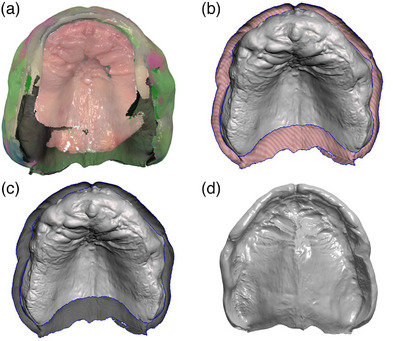
Definitive impression prior to post‐processing (a). Trimming irrelevant regions of scan (b). Inversion (flipping of normal) of IOS portion (light grey) of model with exaggerated junction (blue line) between the scan to demonstrate the different areas (c). Definitive digital model (d). IOS, intraoral scanners.

## DISCUSSION

This technique demonstrates that an intraoral scan and silicone impression can be captured and integrated in situ. This technique aligns more closely to a conventional open tray impression technique where both phases of the impression are made in situ. No additional clinical appointments are required compared to traditional complete denture, five appointment clinical protocols, workflows, and the additional clinical and technical time is minimal. This does contrast with digital denture workflows that incorporate reduced clinical appointments such as the ‘Bouma try‐in’, incorporating four clinical appointments, or the ‘Wagner try‐in’ that incorporates three clinical appointments.^3^ These clinical procedures rely on the patient already having existing dentures and having anatomy that is suitable for mucocompressive techniques, which may not be suitable in all clinical scenarios. The concepts in this proposed technique can be incorporated into denture workflows that minimize clinical appointments.

This is an improvement on previous techniques that rely on the alignment of the IOS and silicone impression in metrology or dental computer‐aided design software. It also has the advantage of allowing the clinician to evaluate the open window area of the custom tray to be captured during the impression making. The evaluation can occur at five time points: prior to the impression, while adjusting the tray, after the impression is taken, while adjusting the impression extensions, and after the IOS has been made. The benefits of combining these two techniques are that the functional and compressive properties of conventional PVS impressions can be combined with the mucostatic nature of IOS. In clinical scenarios where mobile flabby ridges are present this is of considerable clinical advantage as the IOS portion of the impression is mucostatic. For patients with a strong gag reflex the post palatal potion can be with an IOS and not captured with the silicone impression by leaving the posterior position of the tray out of the design. When compared to conventional PVS impressions that are cast in gypsum, and then scanned to commence a digital process, this technique provides several advantages. The proposed technique eliminates the time‐consuming processes of impression disinfection and conventional cast production. Furthermore, the elimination of these steps can minimize possible inaccuracy in the process associated with post impression disinfection changes,^21^ or variables associated with gypsum products dimensional changes in cast manufacturing.^22^

Previous techniques, which aligned scans in a laboratory setting, can be problematic. Intraoral scanning and silicon impressions, although similar in capturing palate details, exhibit differences in level of detail and occasional compression in silicone impressions. Therefore, relying solely on software applications for precise scan alignment may result in misalignment, misfits, and potential post‐insertion complications. The proposed technique mitigates these issues by ensuring correct tray positioning during the alignment scan. This technique could also be modified for completely edentulous mandibles where a narrow mobile ridge can lead to tissue displacement during the conventional making of a silicone impression or in the maxillary where patients have a sensitive gag reflex by excluding the posterior segment of the tray covering the post palatal region. Moreover, this technique can also be utilized to record a vertical dimension by adding wax occlusal rims in the posterior quadrants, if desired.

## SUMMARY

Intraoral scans offer the advantage of data acquisition of the mucosal tissue of the oral cavity with no contact, resulting in a completely mucostatic impression. However, border molding with an intraoral scanner is not possible and still relies on an impression. The technique presented here integrates intraoral scans and silicone impressions in situ, aligning closely with open tray impression methods. It improves upon previous approaches by allowing real‐time adjustment and evaluation of the custom tray's fit and impression extensions, addressing issues associated with alignment software and potential misalignment.
